# Mental Health During the First Weeks of the COVID-19 Pandemic in the United States

**DOI:** 10.3389/fpsyt.2021.561898

**Published:** 2021-04-22

**Authors:** William D. S. Killgore, Sara A. Cloonan, Emily C. Taylor, Natalie S. Dailey

**Affiliations:** Social, Cognitive, and Affective Neuroscience Lab, Department of Psychiatry, University of Arizona College of Medicine, Tucson, AZ, United States

**Keywords:** COVID-19, depression, generalized anxiety, PTSD, insomnia, mental health, job loss, financial worries

## Abstract

**Background:** By March 2020, the World Health Organization declared the COVID-19 crisis as a worldwide pandemic and many local governments instituted stay-at-home orders and closed non-essential businesses. Within the United States, tens of millions of workers lost their jobs and financial security during the first few weeks of the national response, in an attempt to slow the global pandemic. Because of the enormity of the pandemic and its potential impact on mental health, the objective of the present study was to document the prevalence of mental health problems and their association with pandemic-related job loss during the third week of the nationwide shutdown.

**Methods:** Mental health was assessed via online questionnaires among a representative sample of 1,013 U.S. adults on April 9–10, 2020. Rates of clinically significant mental health outcomes were compared between participants who lost their job as a result of COVID-19 restrictions (17.4%) vs. those who did not (82.6%). Bivariate multiple logistic regression identified factors that were predictive of, and protective against, mental health problems.

**Results:** The prevalence of clinically significant symptoms was significantly higher than prior population estimates, ranging from 27 to 32% for depression, 30 to 46% for anxiety disorders, 15 to 18% for acute/post-traumatic stress, 25% for insomnia, and 18% for suicidal ideation. Prevalence estimates were 1.5–1.7 times higher for those who reported job loss due to COVID-19 restrictions than those who did not. Mental health problems were predicted by worry over financial instability, insomnia, social isolation, and alcohol consumption, while getting outside more often, perceived social support, and older age were protective against these problems.

**Conclusions:** During the first 3 weeks of lockdowns/stay-at-home restrictions, mental health problems, including depression, anxiety, insomnia, and acute stress reactions were notably elevated relative to prior population estimates. Job loss related to the nationwide shutdown was particularly associated with poorer mental health. These findings provide a baseline of mental health functioning during the first weeks of the national emergency and lockdown orders in response to COVID-19.

## Introduction

During the first weeks of the COVID-19 pandemic, nations around the globe implemented unprecedented measures to mitigate the transmission of the SARS CoV-2 virus (1, 2). The United States reported the first COVID-19-related death on February 29, 2020, and <2 weeks later, a national state of emergency was called. In response, local and state governments closed schools, shut down all non-essential business, and enacted shelter-in-place orders, with the first state-wide shutdown occurring in California on March 19, 2020. Within a matter of weeks, every state in the country had enacted some form of restriction (3), with most of the U.S. population asked to remain at home and to severely limit physical proximity to others. As a consequence, most employment activities deemed “non-essential” by local governments had ceased or rapidly shifted to remote telecommuting or work-from-home options. This soon led to large scale furloughs and job losses for a large segment of the population (4). Despite the clear public health necessity of the stay-at-home orders and physical distancing strategies to slow the spread of the virus, there is no question that these efforts profoundly altered the basic foundations of the social and occupational lives of much of the population. Consequently, the potential long-term effects of the pandemic and associated restrictions on mental health will likely be a focus of research for years to come.

The financial consequences of the shelter-in-place mitigation strategies were rapidly felt throughout the country. Within the first 6 weeks of state-wide stay-at-home orders in the U.S., more than 33 million Americans had filed new unemployment claims, a level of job loss that had not been seen since the Great Depression (4). This extraordinary surge in unemployment was troubling in light of the well-established findings that that job loss, financial stresses, and lack of social support are leading contributors to suicide, substance abuse, domestic violence, and other mental health issues (5, 6). The rampant uncertainty surrounding the potential course of the pandemic and widespread concerns over financial instability stemming from the lockdown orders led to rising concerns that a surge in mental health problems may be looming on the horizon (7, 8). Fear of the virus, its transmissibility, and its potential lethality contributed to panic and generalized anxiety (9), and raised concerns that post-traumatic stress symptoms could persist long after the pandemic had resolved (10), as has been seen in other countries (11). Quarantines enacted in prior disease outbreaks have also been shown to significantly elevate symptoms of PTSD and depression among the population (12). Moreover, prolonged stay-at-home requirements and social distancing measures may have unintended mental health consequences, as they restrict many of the facets of daily existence that provide emotional resilience, social connection, and satisfaction with life (13). The enormity of the pandemic and its effects on daily existence led many experts to voice concern that that mental health problems may be a lingering issue for years to come (7, 14).

Effective recovery from the pandemic will require a well-documented and comprehensive understanding of the mental health effects that emerged during the acute stages of the crisis. To that end, we studied the point prevalence of mental health problems in a nationally representative sample of adults in the U.S. collected at the very outset of the pandemic, during the third week of the nationwide stay-at-home restrictions. We identified overall rates of mental health concerns in this sample and determined the differences in mental health outcomes between those who had lost their primary employment due to the economic shutdown and those who had not. We believe that these data will be critical for documenting the mental health status of the population during the initial phase of the COVID-19 pandemic and will serve as a benchmark for future research on the long-term psychiatric outcomes of the crisis.

## Methods

### Participants

Here, we summarize mental health data from an online assessment collected over a 28-h period between April 9 and April 10, 2020. This date was selected because it was, at that time, projected to be the peak of expected U.S. deaths due to COVID-19 according to the University of Washington Institute for Health Metrics and Evaluation (IHME; https://covid19.healthdata.org/united-states-of-america) model during the preceding week. Using the Amazon Mechanical Turk (MTurk) online crowdsourcing platform (15), a total of 1,074 participants were screened and provided small financial compensation for their time. All participants were geographically located within the United States (verified by IP address geo-coordinates), were at least 18 years of age, and reported English as their primary language. A brief screen for reading comprehension excluded 48 volunteers from further participation. The remaining 1,026 individuals then completed the online questionnaires. Data from 13 participants were excluded for failing to correctly answer imbedded attention check questions. This resulted in a final sample of 1,013 participants with complete and valid data, which formed the basis of the present analysis. The sampling of participants from each state was closely proportional to state population according to the 2019 U.S. Census, suggesting a nationally representative sample of U.S. adults. Specifically, we calculated the proportion of participants from each state, relative to the national population total for the sample and for the U.S. Census data for each state. The mean absolute difference in sampling proportions and census data proportions across states was 0.004 (i.e., <0.5% point). Further, we found that all sampled state proportions differed from the census data by 1.5% points or less, except for Texas (which was underrepresented in the current sample by 3% points). The intraclass correlation coefficient (ICC) between the sample proportions and the census data across states was quite high (ICC = 0.95, *p* < 0.0001), suggesting that these data are likely representative of the larger population. All participants provided electronic acknowledgment of informed consent after begin provided with a full description of the study. This study was approved by the institutional review board of the University of Arizona.

### Questionnaires and Primary Outcomes

Our goal was to provide a source of reliable documentation of the initial mental health prevalence estimates during the first weeks of the pandemic response, which would provide a benchmark for future research efforts. In an effort to be comprehensive, we included several outcome metrics that assessed similar constructs (e.g., we collected two measures of depressive mood, one longer and more extensive, and another as a brief screener). Outcomes were focused on major mental health symptoms that could potentially result from concerns surrounding the COVID-19 pandemic and/or societal attempts to mitigate the spread of the illness. These included current symptoms (i.e., present within the past week to month) of a major depressive disorder, generalized anxiety disorder (GAD), and acute/post-traumatic stress disorder. Using established instruments, we calculated mean scores and the percentage of the sample exceeding published cut-off scores. Depressive symptoms were assessed with the Beck Depression Inventory-II (≥20) (16) and the Patient Health Questionnaire-9 (≥10) (17). We also examined separately the suicidal ideation scores on each of these depression scales (i.e., Item 9 scores ≥1). Generalized anxiety disorder (GAD) symptoms were measured with the GAD-7 (≥8) (18, 19), the Zung Self-Rated Anxiety Scale (SRAS; ≥36) (20, 21), and the state and trait portions of the Spielberger State-Trait Anxiety Inventory (STAI; ≥46) (22, 23). To assess acute/post-traumatic stress, we administered the National Stressful Events Survey Acute Stress Disorder Short Scale (NSESSS; ≥2) (24, 25), and the Primary Care Post-Traumatic Stress Disorder (PC-PTSD) scale (≥3) (26, 27). We also measured the severity of insomnia symptoms using the Insomnia Severity Index (ISI; ≥15) (28, 29). To examine potential moderators of the mental health outcomes described above, we also collected data on a variety of demographic factors, particularly related to job loss from the shutdown and socioeconomic status, and specific concerns relevant to the ongoing COVID-19 pandemic. These variables are detailed in Table 1.

**Table 1:** Demographic characteristics and COVID-19 concerns of groups of individuals who lost or did not lose their primary employment due to the pandemic.

**Characteristics**	**Total sample**	**COVID-19 job loss**	**No job loss**	***p*-Value**
	**(*N* = 1,013)**	**(*N* = 176)**	**(*N* = 837)**	
^*^Age—yr	36.74 ± 12.09	34.80 ± 10.43	37.14 ± 12.38	0.009
^*^Female sex—no. (%)	567 (56.4)	110 (62.9)	457 (55.0)	n.s.
^*^Education—yr	15.0 ± 2.1	14.6 ± 2.0	15.1 ± 2.1	0.004^†^
^*^Ethnicity—no. (%)				n.s.
White	776 (76.6)	139 (79.0)	637 (76.1)	
Black/African American	99 (9.8)	19 (10.8)	80 (9.6)	
Hispanic/Latino	43 (4.2)	5 (2.8)	38 (4.5)	
Asian	66 (6.5)	8 (4.5)	58 (6.9)	
Native American/American Indian/Alaska Native	5 (0.5)	1 (0.6)	4 (0.5)	
Native Hawaiian/Pacific Islander	1 (0.1)	0 (0.0)	1 (0.1)	
Other	21 (2.1)	4 (2.3)	17 (2.0)	
Prefer not to answer	2 (0.2)	0 (0.0)	2 (0.2)	
^*^Income—no. (%)				0.013
≤ $10,000	50 (4.9)	10 (5.7)	40 (4.8)	
$10,001–$25,000	112 (11.1)	30 (17.1)	82 (9.8)	
$25,001–$50,000	267 (26.4)	53 (30.3)	214 (25.6)	
$50,001–$75,000	238 (23.5)	35 (20.0)	203 (24.3)	
$75,001–$100,000	172 (17.0)	31 (17.7)	141 (16.8)	
$100,001–$150,000	122 (12.1)	11 (6.3)	111 (13.3)	
$150,001–$200,000	34 (3.4)	4 (2.3)	30 (3.6)	
≥$200,001	17 (1.7)	1 (0.6)	16 (1.9)	
^*^COVID-19 related concerns–no. reporting YES (%)				
^*^Have you noticed that you are showing symptoms of COVID-19 (fever, dry cough, fatigue/soreness)?	113 (11.2)	29 (16.5)	84 (10.0)	0.014^†^
^*^Have you been tested for COVID-19?	31 (3.1)	8 (4.5)	23 (2.7)	n.s.
^*^Have you been formally diagnosed with COVID-19?	4 (0.4)	2 (1.1)	2 (0.2)	n.s.
^*^Are you considered to be in a “high-risk” group for COVID-19?	309 (30.5)	57 (32.4)	252 (30.1)	n.s.
^*^Has anyone in your household (i.e., where you live) been diagnosed with COVID-19?	16 (1.6)	7 (4.0)	9 (1.1)	0.005^†^
^*^Have any of your friends, co-workers, or first-degree relatives been diagnosed with COVID-19?	203 (20.0)	47 (26.7)	156 (18.6)	0.015^†^
^*^Do you know anyone personally who has been diagnosed with COVID-19?	344 (34.0)	75 (42.6)	269 (32.1)	0.008^†^
^*^Do you know anyone personally who has died from complications associated with COVID-19?	74 (7.3)	17 (9.7)	57 (6.8)	n.s.
^*^Are you currently “sheltering in place” (i.e., not leaving home except for necessities)?	948 (93.6)	171 (97.2)	777 (92.8)	0.033
^*^Have you become worried about your ability to financially support yourself and loved ones?	555 (54.8)	157 (89.2)	398 (47.6)	<0.001^†^
^*^Do you have someone you care about or who is emotionally close to you that you can talk to daily?	921 (90.9)	162 (92.0)	759 (90.7)	n.s.
^*^Do you feel socially isolated?	579 (57.2)	122 (69.3)	457 (54.6)	<0.001^†^
^*^Do you feel like you have enough social/emotional support to get through this time?	844 (83.3)	128 (72.7)	716 (85.5)	<0.001^†^
^*^Are you engaging in consistent “social distancing” around people (e.g., keeping 6 feet from others)?	977 (96.4)	174 (98.9)	803 (95.9)	n.s.
^*^Are you avoiding all contact with others outside of the home?	865 (85.4)	155 (88.1)	710 (84.8)	n.s.
^*^Do you touch others less?	957 (94.5)	167 (94.9)	790 (94.4)	n.s.
^*^Do you trust other people less?	519 (51.2)	102 (58.0)	417 (49.8)	0.05
^*^How often do you pray?				n.s.
At least once a day	270 (27.4)	49 (29.0)	221 (27.0)	
1–6 days a week	113 (11.4)	19 (11.2)	94 (11.5)	
At least monthly	119 (12.1)	24 (14.2)	95 (11.6)	
Seldom or Never	485 (49.1)	77 (45.6)	408 (49.9)	
^*^Weekly exercise—min	36.2 ± 43.2	36.1 ± 46.3	36.2 ± 42.5	n.s.
^*^Over the past two weeks, how many days did you get outside during sunlight hours for more than 10 minutes?	4.7 ± 2.1	4.8 ± 2.0	4.7 ± 2.1	n.s.
^*^On average, how many minutes did you spend outside in the sunlight each day?	66.0 ± 68.0	68.0 ± 64.3	65.5 ± 68.8	n.s.
^*^How often do you have one drink containing alcohol?—% ≥2 times per wk	25.6%	21.0%	26.5%	n.s.
^*^How many drinks containing alcohol do you have on a typical day when you are drinking?—% ≥3 per session	30.2%	35.7%	29.1%	n.s

† *Significant at false discovery rate (FDR) correction, p <0.05; n.s., non-significant; yr, year; no., number*.

* *Items included as potential predictors of mental health in the binomial logistic regression (as well as job loss category and insomnia severity, which are not listed)*.

### Statistical Analysis

We proposed an initial sample size of 1,000 participants, which would provide 88% power to detect effects of interest at a two-sided significance criterion of α = 0.05, assuming small effect sizes. The data were analyzed using SPSS software (version 26). Means and descriptive statistics were calculated for the sample as a whole, as well as for subgroups of individuals who reported job loss as a result of the societal responses aimed at mitigating the spread of COVID-19. Mean values between job status groups were compared with analysis of covariance (ANCOVA) statistically controlling for pre-pandemic income level, education level, and potential exposure to COVID-19 (i.e., “have you noticed that you are shown symptoms of COVID-19?” and “has anyone in your household been diagnosed with COVID-19?”). For clinically relevant scales where published cut-off points were available, we calculated the percentage of the sample and subgroups that met or exceeded those values. Chi-squared statistics were calculated to compare the percentage of individuals exceeding the cut-offs in each group. Finally, binomial multiple logistic regression analyses, with forward selection using the Likelihood Ratio, were used to identify key concerns, traits, and behavioral factors associated with meeting criteria for a probable mental health issue. Multiple comparisons were controlled by false discovery rate (FDR) adjustment of significance, which was calculated using the Benjamini-Hochberg procedure, as implemented via the online FDR (tool: https://tools.carbocation.com/FDR). The corrections were applied uniformly for all comparisons within each table.

## Results

### Demographics

Primary demographic characteristics of the sample were well-matched to the larger U.S. population (see Methods section). While an upper restriction on age was not set, those who responded to the survey included adults ranging in age from 18 to 82 years and females were slightly over-represented (i.e., 56.4%). Overall, 65.9% of the sample reported a previous year household income of $75,000 or less.

### Overall Mental Health Outcomes

**Figure 1:**
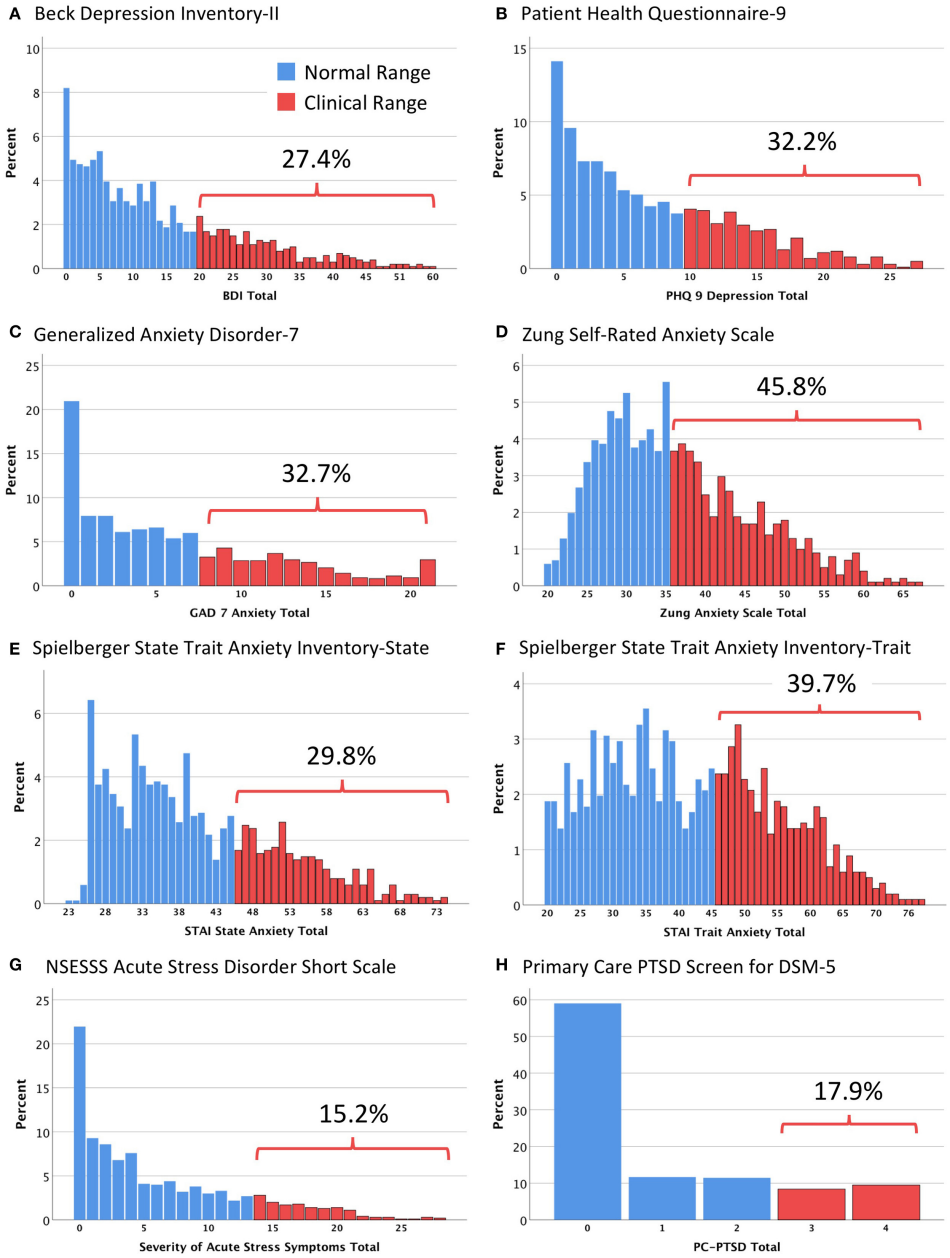
Histograms showing the distribution of scores on the eight major mental health assessment questionnaires. **(A)** Beck Depression Inventory-II (BDI); **(B)** Patient Health Questionnaire-9 (PHQ 9); **(C)** Generalized Anxiety Disorder-7 (GAD 7); **(D)** Zung Self-Rated Anxiety Scale; **(E)** Spielberger State Trait Anxiety Inventory-State (STAI State); **(F)** Spielberger State Trait Anxiety Inventory-Trait (STAI Trait); **(G)** NSESSS Acute Stress Disorder Short Scale; **(H)** Primary Care PTSD Screen for DSM-5 (PC-PTSD). Bars represent the percentage of participants obtaining a particular score. Each histogram divides the sample into those who met published cut-off points for clinical significance (blue, normal range; red, clinical range).

For the sample as a whole, the proportions of individuals screening positive for probable mental health problems was notably higher than would be expected in a similar sample based on prior research (30, 31). For those screening positive for moderate to severe depression (Figure 1, Table 2), the prevalence ranged from 27.2% (BDI-II) to 32.2% (PHQ-9). Across four different scales commonly used to screen for GAD and other clinically significant anxiety disorders, the screen-positive prevalence ranged from 29.8% (STAI-State) to 45.8% (SRAS). Further, 15.2% of the sample screened positive for probable Acute Stress Disorder (NSESSS), while 17.9% screened positive on a brief measure of possible PTSD symptoms (PC-PTSD). Critically, 17.6% of the sample screened positive for some evidence of suicidal ideation on two different scales (BDI-II Item 9; PHQ-9 Item 9), while 25.1% of the sample screened positive for clinically significant insomnia on the ISI.

**Table 2:** Mean scores and percent meeting clinical cut-offs on mental health outcomes between groups of individuals who lost or did not lose their primary employment due to the pandemic.

**Outcome**	**Total sample**	**COVID-19 job loss**	**No job loss**	***p*-Value**
	**(*N* = 1,013)**	**(*N* = 176)**	**(*N* = 837)**	
**BDI**			
Mean total	13.6 ± 12.0	17.5 ± 12.8	12.8 ± 11.7	<0.001^†^
Clinically significant (BDI ≥ 20) {Beck, 1996 #3123}	278 (27.4)	67 (38.1)	211 (25.2)	0.001^†^
**PHQ9**			
Mean total	7.1 ± 6.3	9.2 ± 6.9	6.6 (6.1)	<0.001^†^
Clinically significant (PHQ9 ≥ 10) {Kroenke, 2001 #5911}	362 (32.2)	78 (44.3)	589 (29.6)	<0.001^†^
**GAD7**			
Mean total	6.0 ± 5.8	8.0 ± 6.2	5.6 ± 5.6	<0.001^†^
Clinically significant (GAD7 ≥ 8) {Plummer, 2016 #5914}	321 (32.7)	79 (45.4)	242 (29.9)	<0.001^†^
**Zung SRAS**			
Mean total	36.2 ± 9.4	39.6 ± 10.2	35.5 ± 9.1	<0.001^†^
Clinically significant (SRAS ≥ 36) {Dunstan, 2020 #5915}	462 (45.8)	101 (57.4)	361 (43.3)	0.001^†^
**STAI-state**			
Mean total	40.3 ± 11.0	44.8 ± 11.3	39.3 ± 10.8	<0.001^†^
Clinically significant (STAI-S ≥ 46) {Fisher, 1999 #5916}	302 (29.8)	80 (45.5)	222 (26.6)	<0.001^†^
**STAI-trait**			
Mean total	41.7 ± 13.1	44.8 ± 12.9	41.0 ± 13.1	0.023^†^
Clinically significant (STAI-T ≥ 46) {Fisher, 1999 #5916}	402 (39.7)	84 (47.7)	318 (38.0)	0.016^†^
**NSESSS**			
Mean total	6.1 ± 6.3	7.9 ± 6.8	5.7 ± 6.1	0.001^†^
Clinically significant (SASS ≥ 2) {Kilpatrick, 2013 #5917}	152 (15.2)	40 (22.9)	112 (13.5)	0.002^†^
**PC-PTSD**			
Mean total	1.0 ± 1.4	1.3 ± 1.5	0.9 ± 1.3	0.014^†^
Clinically significant (PC-PTSD ≥ 3) {Ouimette, 2008 #5910}	181 (17.9)	44 (25.0)	137 (16.4)	0.007^†^
**Suicidal ideation (BDI Item 9)**			
Mean total	0.2 ± 0.7	0.2 ± 0.5	0.2 ± 0.5	n.s.
Clinically significant (BDI Item 9 ≥ 1)	178 (17.6)	37 (21.0)	141 (16.8)	n.s.
**Suicidal ideation (PHQ9 Item 9)**			
Mean total	0.3 ± 0.5	0.4 ± 0.8	0.3 ± 0.7	n.s
Clinically significant (PHQ Item 9 ≥ 1)	178 (17.6)	44 (25.0)	134 (16.0)	0.004^†^
**ISI**			
Mean total	9.5 ± 6.8	11.1 ± 6.9	9.2 ± 6.7	0.011^†^
Clinically significant (ISI ≥ 15)	254 (25.1)	57 (32.4)	197 (23.4)	0.014^†^

*n.s., non-significant; yr, year; no., number. BDI, Beck Depression Inventory; PHQ, Patient Health Questionnaire; GAD7, Generalized Anxiety Disorder; SRAS, Self-Rated Anxiety Scale; STAI, State-Trait Anxiety Inventory; NSESSS, National Stressful Events Survey Acute Stress Disorder Short Scale; PC-PTSD, Primary Care Post-traumatic Stress Disorders inventory; ISI, Insomnia Severity Index*.

† *Significant at false discovery rate (FDR) correction, p <0.05; Mean comparisons corrected for prior income, formal educational attainment, and potential exposure to COVID-19 (i.e., personally showing symptoms of COVID-19; someone in household diagnosed with COVID-19)*.

### COVID-19 Job Loss and Mental Health Outcomes

When asked the question “Have you lost your primary job/income due to COVID-19?”, 17.4% of the respondents answered “yes,” while 82.6% answered “no.” As evident in Table 2, individuals who lost their job due to COVID-19 scored significantly higher on measures of depression (BDI-II and PHQ-9). Moreover, 38.1% of those who reported job loss due to COVID-19 exceeded the cut-off for moderate to severe depression on the BDI-II, whereas 25.2% of those who had not lost their job met that criterion. This was even more notable for the PHQ-9, with 44.3% of those who lost their job scoring in the clinically significant range, compared to 29.6% who did not. These differences were significant even after controlling for pre-pandemic income level, education, and the perception of close exposure to COVID-19.

Similar patterns were observed for measures of GAD and other anxiety disorders. Most notably, 57.4% of those who lost their job during the pandemic met the cut-off for a probable anxiety disorder on the Zung SRAS, compared to 43.3% who had not lost their job.

Evidence of probable acute stress and post-traumatic stress reactions was evident in 22.9% and 25.0% of those who lost a job due to the coronavirus outbreak, respectively, compared to 13.5% and 16.4%, respectively, among those whose jobs were not affected.

While suicidal ideation on Item 9 of the BDI-II was not different between job-loss groups, there was a significant difference in suicidal ideation on Item 9 of the PHQ-9, with 25% of those who reported losing their job endorsing some level of suicidal ideation, compared to 16% of those who had not.

Finally, the data in Table 2 also show that 32.4% of those who lost their job met or exceeded the cut-off for clinically significant insomnia on the ISI, while 23.5% of those who had not lost their job met this level of severity.

### Contributing Factors to Mental Health Outcomes

To identify some potential factors that may mitigate or exacerbate mental health problems during the pandemic, we queried participants on a series of questions related to concerns about COVID-19 (see Table 1). While it is acknowledged that no single set of variables will provide a comprehensive explanation of mental health issues, we selected a set of items focused on COVID-19 concerns that we believed would likely play a role in mental health responses to the pandemic. This approach, using background knowledge to aid in variable selection is considered to be an accepted approach to regression analysis (32). For the sample as a whole, a total of 28 potential variables of interest were initially included based on their relevance to pandemic-related concerns at the time, including fears of the virus itself, being in close proximity to someone with the virus, perceived social support, daily activities, alcohol consumption, and basic demographics. The analysis included the asterisked items in Table 1, as well as variables assessing job loss due to the pandemic and insomnia severity. These 28 variables were entered into a binomial multiple logistic regression with forward selection to predict the likelihood of meeting positive screening criteria for each of the 10 mental health outcome variables with published cut-offs (see Table 2). The variables surviving selection for each model are listed in Table 3. The most significant predictors of meeting criteria for moderate to severe depression on the BDI-II and the PHQ-9 included worry about the ability to financially support oneself or family, feeling socially isolated, and greater alcohol use, while spending more days each week outside in the sunshine and feeling that one had enough social support appeared protective against depression. As evident in Table 3, while there were a number of factors associated with screening positive for an anxiety disorder on the GAD-7, Zung SRAS, STAI-S, and STAI-T, the most consistent predictors across measures included endorsing worry about the ability to financially support self or family, feeling socially isolated, and problems with insomnia, while spending more days per week outside in the sunshine appeared most consistently protective. The probability of meeting criteria for acute stress or post-traumatic stress reaction on the NSESSS or PC-PTSD was greatest among those reporting worry about financial problems, feeling socially isolated, trusting others less, and endorsing more problems with insomnia. Finally, screening positive for suicidal ideation on Item 9 of the BDI-II and PHQ-9 was most associated with endorsing financial worries, greater alcohol use, and problems with insomnia, while protective factors against suicidal ideation included male sex, older age, and feeling that one had enough social support to get through the crisis.

**Table 3:** Factors contributing to total sample mental health outcomes based on binomial logistic regression.

	**Nagelkerke *R*^**2**^**	**β (*SE*)**	***p*-Value**	**Adjusted odds ratio (95% CI)**
BDI depression	^†^0.39		<0.001	
Worry about ability to financially support self/family		0.90 (0.19)	<0.001	2.45 (1.70–3.54)
Feel socially isolated		0.50 (0.19)	0.011	1.64 (1.12–2.40)
Alcoholic drinks per day		0.25 (0.10)	0.013	1.28 (1.05–1.55)
Insomnia severity index score		0.13 (0.01)	<0.001	1.13 (1.10–1.17)
Age—yr		−0.03 (0.01)	<0.001	0.97 (0.96–0.99)
Days outside in sunlight per week—no.		−0.10 (0.04)	0.012	0.90 (0.83–0.98)
Have enough social support to get through this		−1.07 (0.21)	<0.001	0.34 (0.22–0.52)
PHQ-9 depression	^†^0.46		<0.001	
Worry about ability to financially support self/family		0.60 (0.18)	0.001	1.83 (1.28–2.61)
Feel socially isolated		0.58 (0.19)	0.003	1.78 (1.23–2.60)
Alcoholic drinks per day		0.35 (0.10)	<0.001	1.42 (1.17–1.73)
Insomnia severity index score		0.19 (0.02)	<0.001	1.20 (1.17–1.24)
Days outside in sunlight per week—no.		−0.18 (0.04)	<0.001	0.84 (0.77–0.91)
Touch others less		−0.78 (0.36)	0.029	0.46 (0.23–0.92)
Have enough social support to get through this		−0.81 (0.23)	<0.001	0.45 (0.29–0.69)
GAD-7 generalized anxiety disorder	^†^0.42		<0.001	
Worry about ability to financially support self/family		0.84 (0.18)	<0.001	2.33 (1.64–3.30)
Feel socially isolated		0.76 (0.18)	<0.001	2.13 (1.49–3.05)
Female sex		0.56 (0.17)	0.001	1.75 (1.25–2.47)
Trust others less		0.41 (0.17)	0.017	1.51 (1.08–2.12)
Insomnia severity index score		0.16 (0.01)	<0.001	1.17 (1.14–1.21)
Age—yr		−0.03 (0.01)	<0.001	0.97 (0.96–0.99)
Days outside in sunlight per week—no.		−0.11 (0.04)	0.005	0.89 (0.82–0.97)
Anyone in household been diagnosed with COVID-19		−1.54 (0.65)	0.019	0.21 (0.06–0.78)
Zung SRAS anxiety disorder	^†^0.47		<0.001	
Worry about ability to financially support self/family		0.81 (0.16)	<0.001	2.24 (1.62–3.09)
Trust others less		0.70 (0.16)	<0.001	2.02 (1.46–2.78)
Female sex		0.50 (0.17)	0.002	1.65 (1.20–2.29)
Know someone personally diagnosed with COVID-19		0.38 (0.17)	0.026	1.46 (1.05–2.03)
Feel socially isolated		0.36 (0.17)	0.031	1.44 (1.03–2.00)
Alcoholic drinks per day		0.22 (0.10)	0.026	1.25 (1.03–1.52)
Insomnia severity index score		0.18 (0.01)	<0.001	1.20 (1.16–1.23)
Days outside in sunlight per week—no.		−0.15 (0.04)	<0.001	0.86 (0.80–0.93)
Touch others less		−0.98 (0.35)	0.005	0.38 (0.19–0.74)
STAI-state anxiety disorder	^†^0.36		<0.001	
Worry about ability to financially support self/family		1.06 (0.18)	<0.001	2.87 (2.03–4.08)
Feel socially isolated		0.73 (0.19)	<0.001	2.07 (1.44–2.98)
Female sex		0.48 (0.17)	0.005	1.61 (1.15–2.24)
Insomnia severity index score		0.13 (0.01)	<0.001	1.14 (1.11–1.17)
Days outside in sunlight per week—no.		−0.10 (0.04)	0.008	0.90 (0.84–0.97)
High risk for COVID-19		−0.39 (0.18)	0.035	0.68 (0.48–0.97)
Have enough social support to get through this		−0.44 (0.21)	0.037	0.64 (0.43–0.97)
STAI-trait anxiety disorder	^†^0.35		<0.001	
Worry about ability to financially support self/family		0.68 (0.16)	<0.001	1.97 (1.44–2.68)
Feel socially isolated		0.67 (0.17)	<0.001	1.96 (1.42–2.71)
Prayer frequency		0.22 (0.06)	<0.001	1.25 (1.11–1.40)
Insomnia severity index score		0.10 (0.01)	<0.001	1.11 (1.08–1.14)
Min of exercise per week		−0.01 (0.00)	0.008	0.99 (0.99–1.00)
Age—yr		−0.02 (0.01)	0.009	0.98 (0.97–1.00)
Days outside in sunlight per week—no.		−0.14 (0.04)	<0.001	0.87 (0.81–0.94)
Touch others less		−0.71 (0.32)	0.026	0.49 (0.26–0.92)
Have enough social support to get through this		−0.75 (0.22)	0.001	0.47 (0.31–0.72)
NSESSS acute stress disorder	^†^0.34		<0.001	
Been tested for COVID-19		1.15 (0.45)	0.010	3.17 (1.32–7.59)
Trust others less		0.80 (0.23)	<0.001	2.22 (1.42–3.49)
Worry about ability to financially support self/family		0.75 (0.24)	0.002	2.11 (1.32–3.39)
Feel socially isolated		0.69 (0.25)	0.005	2.00 (1.23–3.27)
Insomnia severity index score		0.14 (0.02)	<0.001	1.15 (1.11–1.19)
Age—yr		−0.05 (0.01)	<0.001	0.96 (0.94–0.98)
Prayer frequency		−0.18 (0.08)	0.030	0.84 (0.71–0.98)
Currently sheltering in place		−0.79 (0.38)	0.038	0.45 (0.21–0.96)
PC-PTSD screen positive	0.28		<0.001	
Feel socially isolated		0.74 (0.22)	0.001	2.10 (1.36–3.23)
Worry about ability to financially support self/family		0.70 (0.21)	0.001	2.01 (1.33–3.06)
Showing symptoms of COVID-19		0.69 (0.25)	0.005	1.99 (1.23–3.23)
Know someone personally who died from COVID-19		0.61 (0.31)	0.049	1.84 (1.00–3.37)
Trust others less		0.51 (0.20)	0.011	1.66 (1.12–2.46)
Insomnia severity index score		0.11 (0.01)	<0.001	1.12 (1.09–1.15)
BDI suicidal ideation	^†^0.23		<0.001	
Worry about ability to financially support self/family		0.64 (0.20)	0.002	1.89 (1.27–2.81)
Alcoholic drinks per day		0.33 (0.10)	0.001	1.38 (1.14–1.68)
Insomnia severity index score		0.07 (0.01)	<0.001	1.07 (1.04–1.10)
Average minutes outside in sunlight per day—no.		0.00 (0.00)	0.021	1.00 (0.99–1.00)
Age—yr		−0.02 (0.01)	0.012	0.98 (0.96–1.00)
Female sex		−0.54 (0.19)	0.004	0.58 (0.40–0.84)
Someone emotionally close…can talk to daily		−0.63 (0.27)	0.021	0.53 (0.31–0.91)
Touch others less		−0.82 (0.35)	0.018	0.44 (0.22–0.87)
Have enough social support to get through this		−0.89 (0.22)	<0.001	0.41 (0.27–0.63)
PSQ-9 suicidal ideation	^†^0.26		<0.001	
Worry about ability to financially support self/family		0.55 (0.21)	0.007	1.74 (1.16–2.61)
Alcoholic drinks per day		0.31 (0.10)	0.002	1.37 (1.12–1.67)
Insomnia severity index score		0.09 (0.01)	<0.001	1.09 (1.06–1.13)
Age—yr		−0.03 (0.01)	0.004	0.97 (0.96–0.99)
Days outside in sunlight per week—no.		−0.15 (0.04)	0.001	0.86 (0.79–0.94)
Female sex		−0.50 (0.19)	0.009	0.61 (0.42–0.88)
Touch others less		−0.77 (0.36)	0.032	0.46 (0.23–0.93)
Have enough social support to get through this		−0.96 (0.21)	<0.001	0.38 (0.25–0.58)

† *Significant at false discovery rate (FDR) correction, p <0.05; n.s., non-significant; yr, year; no., number*.

## Discussion

The reported prevalence of mental health problems during the first weeks of the pandemic response in the U.S. was notably higher than expected based on general population estimates collected over the decade prior to the pandemic. Prior research has estimated that the 12-month prevalence for mental health problems in the general population to be approximately 9.3% for any major depressive episode, 2.9% for GAD, and 4.4% for PTSD (30), while reported suicidal ideation ranges from 2.0 to 3.7% (31). Our findings suggest that the prevalence of probable mental health problems at this early phase of the pandemic was higher than estimates from prior years. Consistent with other contemporaneous research (33), we found that major depression was 2.9–3.5 times higher than before the pandemic; GAD 10.3–15.8 times higher; and acute-stress/post-traumatic stress 3.5–4.0 times higher. Moreover, suicidal ideation was found to be 4.8–8.8 times higher than prior population estimates. It should be kept in mind that brief screening methods such as those used here may tend to overestimate mental health prevalence rates relative to gold-standard clinical interviews (34), and that the comparison data were, in many cases, collected years earlier. Nonetheless, with due consideration to this risk, the magnitude of the findings raise serious concerns about the mental health status of the general U.S. population during the early phase of the COVID-19 pandemic.

As a result of necessary and vital efforts to slow the spread of the novel coronavirus, non-essential businesses were closed and tens of millions of Americans found themselves out of work. Consistent with the unemployment data from the first month of the shutdown restrictions, 17.4% of the participants in this study reported having lost their primary job as a direct result of the COVID-19 pandemic. While mental health problems were notably high for the sample as a whole, those who lost their primary job directly as a result of the pandemic consistently showed greater severity across all measures of depression, anxiety, and stress responses. The prevalence of clinically significant mental health problems was 1.5–1.7 times higher among those who reported a COVID-19-related job loss than those who did not report such a loss. Large meta-analyses have shown that mental health problems are about twice as prevalent among individuals who are unemployed than those who are employed (35). Our findings suggest that the difference in the rates of mental health problems between those who did and did not lose their jobs appears to be very similar to those of prior studies, but perhaps slightly smaller in the magnitude of difference, probably due to elevated rates of general pandemic-related concerns even among those who did not experience a COVID-related job loss. Unemployment and financial insecurity are well known contributors to poorer mental health, including depression and suicide (6). Our data are consistent with the existing literature and suggest that the rise in unemployment during the pandemic is associated with significantly elevated mental health problems.

For the sample as a whole, poorer mental health outcomes tended to be predicted by greater worry about the ability to financially support oneself or loved ones, feeling socially isolated, greater severity of insomnia symptoms, and consuming more alcohol. On the other hand, consistent with prior research, protective factors included spending more days per week outside in the sunlight, perceiving enough social and emotional support to get through the crisis, and older age. The contributory role of each of these factors is not surprising and all have been supported by considerable research (13, 36–43). In particular, numerous studies suggest that younger age groups have exhibited greater mental health problems as a result of the pandemic (40–43). Ways to encourage safe outside activities and facilitate social and emotional support need to be explored and encouraged to help individuals maintain resilience and wellbeing during the pandemic stay-at-home period.

How can these data inform psychiatric care and public health policy? The data suggest that subjective reports of financial worry represented the most consistent and predictive factor associated with meeting criteria for clinically significant mental health problems. Because of the extraordinarily high level of job loss produced as a direct consequence of the pandemic response, these findings suggest that efforts to address the personal financial impacts of the pandemic are going to be pivotal contributors to averting an impending mental health crisis. Social isolation and a sense of insufficient social support each also contributed significantly to mental health problems. This is consistent with other data suggesting that loneliness has increased during the course of the pandemic, and is associated with suicidal ideation and other mental health issues (36, 44, 45). Clearly, any successful psychiatric mitigation strategy will need to address the profound issues surrounding the current reduction in face-to-face human interaction and the widespread experience of loneliness (46). Problems with insomnia were also highly predictive of poorer mental health, suggesting that sleep assessment should be incorporated into routine clinical contacts and behavioral and medical efforts aimed toward facilitating better sleep health should be a priority (47). Further, suicidal ideation was predicted by greater alcohol intake in combination with financial worries. This is particularly concerning, as recent evidence suggests that alcohol purchases, consumption, and dependence behaviors increased dramatically for those under lockdown during the first 6-months of the pandemic (39, 48, 49). For those at risk of suicidal ideation, alcohol intake should be minimized/avoided (5). Finally, spending more days outside in sunlight was frequently a predictor of positive mental health outcomes. Light exposure is important for enhancing mood and maintaining a healthy sleep schedule (50). Even during prolonged stay-at-home mandates, it is recommended that individuals find ways to increase daylight exposure and, when appropriate, to engage safely and responsibly in appropriately socially distanced outdoor activities to maintain mental health and wellbeing.

A small, but interesting finding is also worthy of note. In Table 3, we found that scores on the GAD-7 were lower among individuals who also reported that they lived with someone in the household who had been diagnosed with COVID-19. We interpret this counterintuitive finding as the effect of seeing COVID-19 first-hand, which may have reduced anxiety over the unknown. During the early weeks of the pandemic, not much was known about the virus, which led to much speculation and widespread worry. Since the vast majority of people who contract COVID-19 tend to be asymptomatic or experience only mild illness, the experience of direct exposure to someone who has been diagnosed and potentially recovered may actually have reduced their anxiety by making the illness concrete. Of course, this is *post-hoc* speculation, and will require further research.

While the present sample was collected to be representative of the general U.S. population, it is important to keep in mind that the data may not be representative of the mental health responses in other areas of the world. COVID-19 has affected every country on the globe, but the response to the pandemic has been different across cultures. For instance, cultures that adhere to tightly to social norms appear to have fared much better with regard to the number of COVID-19 cases and deaths than cultures that adhere much more loosely to such norms (such as the U.S.) (51). Consequently, the willingness to accept the necessity or legitimacy of the government lockdowns and their repercussions on social or occupational functioning may play a role in how job loss may be perceived and how it may affect mental health. The present research does not directly address this issue, but it will be one that is important for further study. In the meantime, the generalizability of these findings to other cultures with different values should be considered as tentative until validated with further research in other countries around the world.

This study was limited by its use of self-report measures and online questionnaires rather than in-person clinical interviews. Future work will involve more extensive clinical interviews and longitudinal data collection to monitor changes during the course of the pandemic. Furthermore, at the time when these data were first collected, there were no readily available validated COVID-19 metrics to assess mental health issues, which is a clear limitation. Since that time, validated metrics such as the Fear of COVID-19 Scale have become available (52), and are recommended for use in future COVID-19-related studies. The present findings are also limited by the fact that most of the large epidemiological samples to which we compared our findings were collected some time ago and may differ somewhat from the demographics of the current sample. Population prevalence rates for various disorders change over time and so it is possible that our findings overestimate the prevalence of these mental health issues. Additionally, the questionnaires we used were generally designed for screening purposes rather than comprehensive psychodiagnostic assessment. Such metrics are often designed for high sensitivity relative to specificity, and may lead to an overestimation of the prevalence of certain disorders (34). These limitations notwithstanding, the present findings strongly suggest that the U.S. population experienced extraordinary mental health concerns in the first weeks after nationwide pandemic restrictions were enacted (14). It is conceivable that these mental health problems will persist or even increase in the coming months and years as the long-term occupational and personal financial fallout from the pandemic continues to be realized. Large scale efforts to mitigate the effects of financial instability and facilitate social connectedness will be crucial to minimizing the long-term impact of the pandemic on mental health.

## Data Availability Statement

The raw data supporting the conclusions of this article will be made available by the authors, without undue reservation.

## Ethics Statement

The studies involving human participants were reviewed and approved by University of Arizona Institutional Review Board. The patients/participants provided their electronic informed consent to participate in this study.

## Author Contributions

WK primary study design and conceptualization, primary literature search, data analysis, data interpretation, writing of the initial draft, and figures and tables. SC contributed to study design, data collection, and editing drafts of manuscript. ET and ND contributed to study design and editing drafts of manuscript. All authors contributed to the article and approved the submitted version.

## Conflict of Interest

The authors declare that the research was conducted in the absence of any commercial or financial relationships that could be construed as a potential conflict of interest.
